# Endocrine and Metabolic Challenges of Long-Term Lithium Therapy: Diagnostic and Management Considerations

**DOI:** 10.7759/cureus.88526

**Published:** 2025-07-22

**Authors:** Stylianos Kopanos, Joachim Feldkamp

**Affiliations:** 1 Academic Department of Endocrinology, Diabetes and Infectiology, Klinikum Bielefeld, Medical School and University Medical Centre East Westphalia-Lippe Bielefeld University, Bielefeld, DEU

**Keywords:** calcimimetics, hypercalcemia, lithium-induced hyperparathyroidism, nephrogenic diabetes insipidus, osteoporosis

## Abstract

Lithium is a key treatment for bipolar disorder but is associated with significant endocrine and metabolic side effects, particularly lithium-induced hyperparathyroidism (LIH). Characterized by hypercalcemia and inappropriately normal or elevated parathyroid hormone (PTH) levels, LIH can lead to skeletal complications such as osteoporosis. Managing this condition is particularly challenging in psychiatric patients due to adherence issues with follow-up care. We describe the case of a 56-year-old Caucasian female patient with bipolar disorder on long-term lithium therapy (16 years), presenting with recurrent hypercalcemia, hyperparathyroidism, and osteoporosis. Symptoms included fatigue, depression, and cognitive difficulties. Laboratory tests confirmed hypercalcemia (2.8 mmol/L), normal PTH (47.5 pg/mL), and vitamin D deficiency (11.8 ng/mL). Imaging identified a parathyroid adenoma and osteoporosis. Parathyroidectomy failed to resolve hypercalcemia, which persisted at 2.6 mmol/L. Lithium discontinuation led to calcium normalization and improved bone density (8.2% increase in lumbar spine measurements). However, follow-up imaging revealed a small epithelial adenoma, raising concerns about further surgical intervention. Management was complicated by poor adherence to follow-up care. This case highlights the complexities of diagnosing and managing LIH, particularly in patients with psychiatric comorbidities. A multidisciplinary approach involving endocrinology and psychiatry is essential to optimize treatment while balancing lithium’s therapeutic benefits against its risks. Further research is needed to refine surgical strategies, calcimimetic therapy, and long-term follow-up protocols for effective management.

## Introduction

Lithium is commonly used for managing acute mania and as a maintenance therapy for bipolar disorder, despite its narrow therapeutic range (0.6-1.2 mM) with hypercalcaemia observed in 3-30% of patients [1]. However, it is associated with endocrine and metabolic side effects, affecting thyroid, calcium metabolism, water and electrolyte balance, and nephrogenic diabetes insipidus.

The primary endocrine effects of lithium involve the kidneys, thyroid, parathyroid glands, pancreas, and the hypothalamic-pituitary-adrenal axis. Its mechanisms include inhibition of thyroid-stimulating hormone (TSH)-sensitive adenylate cyclase, leading to hypothyroidism; reduced aquaporin-2 expression, contributing to nephrogenic diabetes insipidus; and disruptions in calcium homeostasis, resulting in hyperparathyroidism and hypercalcemia, by interfering with transmembrane signal transduction in calcium-sensing receptors (CaSR). This increases the functional threshold of the parathyroid gland, enhances renal calcium reabsorption at the loop of Henle, and directly stimulates parathyroid hormone (PTH) release [2].

Albert et al. highlight that chronic lithium use may exacerbate pre-existing subclinical parathyroid pathology, resulting in recurrent parathyroid adenomas, parathyroid hyperplasia, and multiple adenomas [3]. Approximately 50% of patients with lithium-induced hyperparathyroidism (LIH) who undergo surgery exhibit multiple parathyroid lesions, compared to fewer cases in primary hyperparathyroidism.

The endocrine side effects of lithium therapy include thyroid dysfunction (goiter, hypothyroidism, or occasionally hyperthyroidism), hyperparathyroidism (hypercalcemia and bone demineralization), hyperglycaemia, water-sodium imbalance, nephrogenic diabetes insipidus, and generalized symptoms such as fatigue and neuropsychiatric disturbances (e.g., depression, delirium, catatonia). These symptoms may exacerbate underlying mood disorders [4].

Currently, there are no standardized guidelines for screening or managing lithium-induced calcium disorders. Lithium discontinuation should be considered in consultation with a psychiatrist, as it may reduce hypercalcemia, particularly in atypical cases. Multidisciplinary monitoring is crucial to address potential symptoms of lithium withdrawal, such as the rebound of the underlying condition or mood disorders. Annual serum calcium monitoring or testing after an intercurrent event, such as mood disorder relapse, is advised since hypercalcemia can exacerbate mood disorders. 

Surgical intervention is being considered, particularly for bone or renal complications. Kovacs et al. suggest that surgery is typically indicated for hyperparathyroidism with a solitary adenoma, whereas calcimimetic agents may be preferred in other cases [5].

We report the case of a 56-year-old female patient with schizoaffective disorder, on long-term lithium therapy, who presented with resistant hypercalcemia, recurrent parathyroid adenomas with dysplastic histological features, and mild osteoporosis.

## Case presentation

A 56-year-old Caucasian woman was referred to our endocrinological outpatient clinic for the management of recurrent hypercalcemia diagnosed six months ago, and presented with symptoms including hyperalgesia, apathy, aboulia, asthenia, concentration difficulties, polyphagia, and nausea.

Her medical history revealed a mixed schizoaffective disorder, for which she had been on lithium therapy for 16 years. She also had a history of endometriosis treated with a Mirena spiral, recurrent urolithiasis complicated by haematuria and kidney stones requiring double-J stent placement for urinary drainage, and cervical spine stenosis and osteoporosis. Hypothyroidism, diagnosed five years earlier without evidence of autoimmunity or goiter, was managed with levothyroxine. Recent cardiological evaluations showed no abnormalities. Additional symptoms included confusion, dyspnoea, generalized abdominal pain, libido loss, and anxiety. The patient’s BMI was 31.4 kg/m², indicating class I obesity.

Her medications consisted of levothyroxine, lithium, and non-steroidal anti-inflammatory drugs prescribed for fibromyalgia-like symptoms. Both physical and psychomotor examinations were unremarkable, and there was no reported family history of hypercalcemia.

Investigation

**Figure 1:**
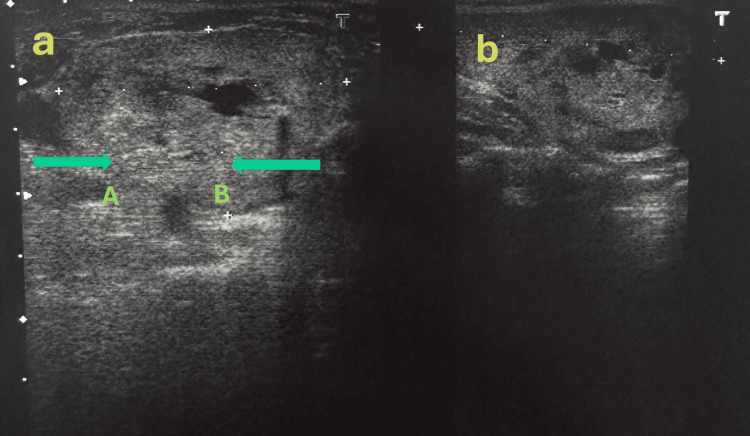
Ultrasonography showing parathyroid adenoma in the lower left region (green arrows delineate the respective zones evaluated in both axes) (a) Transverse ultrasound image showing two adjacent regions of interest: (A) a hypoechoic area suggestive of parathyroid adenoma and (B) a comparatively isoechoic or hyperechoic area, possibly representing surrounding tissue and normal parenchyma; (b) Corresponding longitudinal view of the same anatomical structures.

Laboratory evaluations revealed corrected calcium levels of 2.8 mmol/L, PTH of 274 pg/mL, and vitamin D at 11.3 ng/mL (Table 1). Neck ultrasound identified a parathyroid adenoma in the lower left region, measuring 4.4 x 7.2 x 8.3 mm, with predominant peripheral perfusion and a central vascularisation (Figure 1). Thyroid and parathyroid scintigraphy revealed no evidence of autonomous nodes. Dual-energy X-ray absorptiometry (DEXA) indicated significant bone density loss, with L1-L4 measurements at 0.751 g/cm² (T-score: -2.7, Z-score: -1.7) and hip-area measurements at 0.672 g/cm² (T-score: -2.2, Z-score: -1.6), consistent with osteoporosis.

**Table 1:** Laboratory test results TSH: thyroid-stimulating hormone; fT3: free triiodothyronine; fT4: free thyroxine; TPO: thyroid peroxidase; Parathormone: parathyroid hormone (PTH)

Parameter	Patient Value	Reference Range
Calcium	2.8 mmol/L	2.2–2.55 mmol/L
Phosphorus	3.8 mg/dL	2.7–4.5 mg/dL
Vitamin D	11.3 ng/mL	>20 ng/mL
TSH	1.89 μE/mL	0.27–4.2 μE/mL
fT3	2.5 pg/mL	2.0–4.4 pg/mL
fT4	1.4 ng/mL	0.9–1.7 ng/mL
24-hour Urine Calcium	6.7 mmol/24 h	2.5–8 mmol/24 h
TPO Antibody	8 U/L	≤35 U/L
TSH Receptor Antibody	0.30 U/L	≤1.75 U/L
Thyroglobulin Antibody	12 U/L	≤115 U/L
Calcitonin	<10 ng/mL	≤12 pg/mL
Parathormone	274 pg/mL	15–65 pg/mL
Urinary Desoxypyridinoline	6.5 mmol/Crea	3.0–7.4 mmol/Crea

Treatment

Given the fact that the patient's body weight had remained consistent over time, with no significant fluctuations that might impact thyroid hormone dosing or metabolic assessment, levothyroxine therapy was continued at 75 μg/day. Vitamin D supplementation at 1.000 IU/day was initiated to address deficiency and reduce the risk of osteoporotic fractures. 

At the time of surgical decision-making, psychiatric consultation was obtained. The patient was assessed as not psychiatrically stable enough to discontinue lithium therapy or transition safely to an alternative such as an atypical antipsychotic, given her history of mood instability and complex psychiatric comorbidity. Therefore, a surgical approach was prioritized, in line with recommendations for cases involving significant hypercalcemia and parathyroid gland enlargement. Surgery was based on persistent symptomatic hypercalcemia, confirmed adenoma on ultrasound, and progressive bone loss. Scintigraphy was negative, but parathyroid ultrasound revealed a structurally abnormal gland. Following multidisciplinary evaluation, the patient underwent a three-quarter parathyroidectomy (left superior and inferior, and right inferior glands). Histopathological analysis revealed hyperplasia with dysplastic elements in situ. No lymph node involvement or evidence of metastasis was documented.

**Figure 2:**
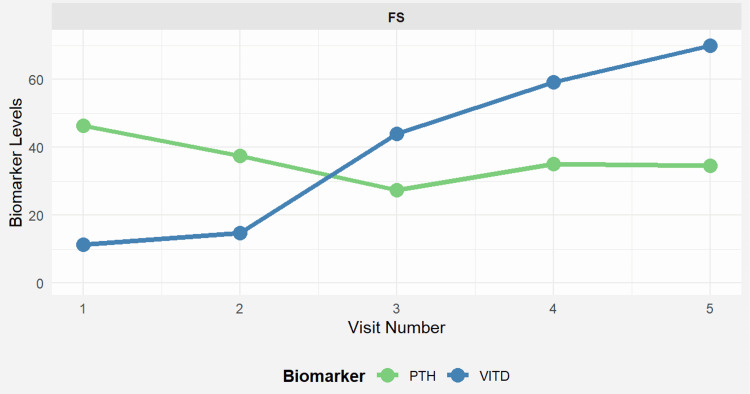
Longitudinal progression of PTH and vitamin D levels over time Visit 1: Initial presentation before parathyroid surgery; Visit 2: One month post-parathyroidectomy; Visit 3: Follow-up prior to lithium discontinuation (hypercalcemia persisted); Visit 4: Three months after lithium discontinuation; Visit 5: Last available follow-up prior to patient’s loss to follow-up PTH values remained elevated despite surgery (Visits 2–3) but normalized following lithium discontinuation (Visit 4). Vitamin D deficiency was treated throughout, with gradual improvement. PTH: parathyroid hormone; VITD: vitamin D

**Figure 3:**
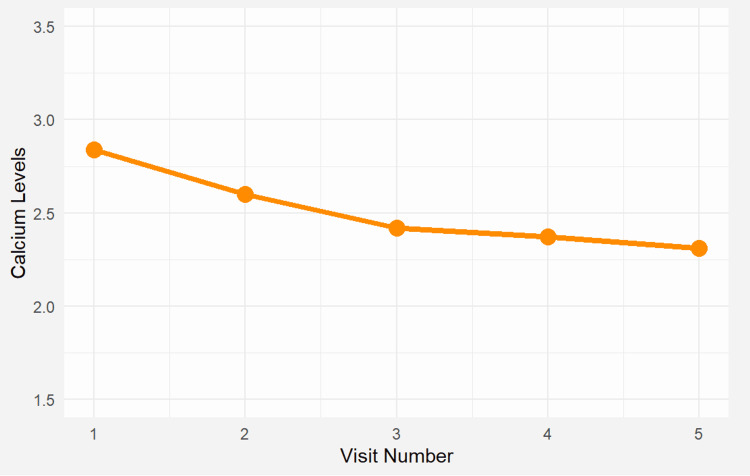
Longitudinal progression of serum calcium progression over time Visit 1 (Month 0): Before parathyroid surgery; Visit 2 (Month 1): Follow-up after parathyroidectomy; Visit 3 (Month 3): Persistent hypercalcemia prior to lithium discontinuation; Visit 4 (Month 4): One month after lithium discontinuation; Visit 5 (Month 5): Last available follow-up before patient was lost to follow-up; Calcium levels remained elevated after surgery (Visit 2–3), but normalized following lithium discontinuation (Visit 4).

Five months later, the patient presented with persistent hyperparathyroidism, accompanied by depression, weakness, and hypercalcemia (2.6 mmol/L) despite normal PTH levels (37.5 pg/mL) and persistent vitamin D deficiency (14.8 ng/mL). Neck ultrasound revealed hypoechoic thyroid parenchyma, with no evidence of additional parathyroid adenomas or disease progression. Renal ultrasound, which was conducted to evaluate for recurrent nephrolithiasis in the setting of ongoing hypercalcemia, excluded nephrolithiasis. Stone analysis at the time of urolithiasis revealed calcium composition. Due to recurrent and therapy-resistant hyperparathyroidism, discontinuation of lithium therapy was recommended following psychiatric consultation (Figures 2, 3).

Outcome and follow-up

Over time, and notably after parathyroid surgery but before lithium was discontinued, the patient developed interstitial tubular nephrosclerosis. This initially presented as acute kidney injury and later progressed to chronic kidney disease, as evidenced by microalbuminuria of 50 μg/minute (reference range: <30 μg/minute) and mildly elevated serum creatinine levels of 1.3 mg/dL. To address progressive renal damage, hypertension, and renal vascular injury, valsartan 160 mg was prescribed to reduce proteinuria, control blood pressure, and improve renal function. Nonsteroidal anti-inflammatory drugs (NSAIDs) were discontinued after nephropathy was identified. Daily diuresis was over 3.2 L/day, suggestive of partial nephrogenic diabetes insipidus. Electrolyte levels (sodium: 138 mmol/L, reference range: 135-145 mmol/L; potassium: 3.8 mmol/L, reference range: 3.5-5.2 mmol/L) and HbA1c (5.8%, reference range: <6.5%) remained within normal limits. Lithium was replaced with duloxetine 60 mg, effectively managing depressive symptoms, fibromyalgia, and neuropathic pain. The patient did not experience any triggering of manic or hypomanic episodes, nor did they report side effects such as dry mouth, dizziness, or insomnia.

Treatment with cinacalcet was considered, but not initiated, because serum calcium levels normalized following lithium withdrawal, and PTH levels remained within range with sustained vitamin D correction. Thus, pharmacological intervention with calcimimetics was deemed unnecessary at that point. Vitamin D supplementation was continued, and five months after surgery, the patient’s PTH level had stabilized at 34.6 pg/mL, and vitamin D levels had risen to 70 ng/mL with ongoing supplementation. A follow-up DEXA scan performed eight months after the initial scan demonstrated an 8.2% improvement in lumbar spine bone mineral density and a 1.1% increase in the hip area, consistent with clinical improvement in osteopenia. A follow-up neck ultrasound performed five months after surgery showed no signs of parathyroid adenomas or new thyroid nodules. However, a subsequent ultrasound at eight months post-surgery identified a small parathyroid adenoma measuring 3.5 x 1.6 x 3.4 mm. This was described as an epithelial parathyroid adenoma, with no thyroid involvement, and thus no thyroid fine-needle aspiration (FNA) was indicated. Although the patient remained asymptomatic with normalized laboratory values and improved bone mineral density, this finding raised the possibility of future surgical re-intervention in the event of clinical or biochemical deterioration. As such, surgical re-intervention was not undertaken, due to the patient’s stable condition, and a conservative, watchful waiting strategy was chosen, with the recommendation for ongoing monitoring in case of future biochemical relapse. Follow-up care was discontinued after the patient’s non-attendance. The last clinical and biochemical follow-up, which occurred five months after lithium discontinuation, before the patient’s loss to follow-up. 

## Discussion

The hallmark biochemical abnormality in LIH is hypercalcemia, resulting from increased PTH secretion. This mirrors the profile seen in primary hyperparathyroidism. Serum phosphate levels are typically low to low-normal, although this finding is more variable in lithium-related cases. A distinguishing feature is that urinary calcium excretion is often inappropriately low or normal despite elevated serum calcium, resembling familial hypocalciuric hypercalcemia (FHH) and helping to differentiate LIH from classical primary forms. Other electrolytes, such as sodium, potassium, and magnesium, are generally unaffected by the hyperparathyroid state itself. However, lithium’s renal effects, particularly its impact on aquaporin-2 expression, can result in nephrogenic diabetes insipidus, contributing to disturbances in sodium and water balance independent of parathyroid function.

Studies suggest that hypercalcemia occurs in 8-15% of patients receiving lithium for bipolar disorder, while elevated PTH levels without concurrent hypercalcemia have been reported in 15-47% of cases. Hypercalcemia is associated with neuropsychiatric symptoms such as delirium and depression, as well as somatic manifestations including constipation and bone pain [6].

Lithium exerts its effects through the inhibition of glycogen synthase kinase-3 (GSK-3), which disrupts cyclic AMP (cAMP) signalling and G-proteins (Table 2). GSK-3 is critical for cell signalling, and its inhibition may contribute to cell cycle arrest, potentially leading to dysplastic developments, as observed in our case. While there have been associations of lithium with medullary thyroid carcinoma, pheochromocytoma, and carcinoid tumors, the evidence remains limited and inconclusive.

**Table 2:** Molecular pathways and pathophysiological mechanisms of lithium CaSR: calcium-sensing receptor; PTH: parathyroid hormone

Molecular Pathway/Effect	Mechanism	Impact
Inhibition of CaSR	Lithium directly interferes with the CaSR in parathyroid cells, reducing its sensitivity to extracellular calcium levels.	Diminished feedback inhibition of PTH secretion, leading to elevated PTH levels even in normocalcemia.
Increased PTH Secretion	Impaired CaSR signalling results in overproduction of PTH despite adequate or elevated serum calcium levels.	Hypersecretion of PTH drives hypercalcemia and secondary metabolic disturbances.
Altered Intracellular Calcium Signalling	Lithium modulates calcium signalling pathways within parathyroid cells, disrupting normal cellular homeostasis.	Further promotes inappropriate PTH release and gland hyperplasia.
Multiglandular Involvement	Chronic stimulation of parathyroid cells leads to compensatory proliferation of multiple glands.	Increases the likelihood of multiglandular hyperplasia, complicating treatment strategies.
Elevated Serum Calcium Levels	Excess PTH increases calcium reabsorption in the kidneys, bone resorption by osteoclasts, and intestinal absorption.	Results in hypercalcemia, with potential for severe clinical consequences like nephrocalcinosis and arrhythmias.
Alteration of Vitamin D Metabolism	Lithium indirectly affects vitamin D by modulating PTH and calcium feedback loops, altering 1α-hydroxylase activity in the kidney.	Disrupts calcium absorption in the gut and amplifies disturbances in calcium-phosphorus balance.
Parathyroid Cell Proliferation	Prolonged PTH overproduction and disrupted cellular signalling stimulate parathyroid cell growth and hyperplasia.	Contributes to the development of primary or secondary hyperparathyroidism.
Reduced Apoptosis in Parathyroid Cells	Lithium may inhibit apoptosis pathways in parathyroid cells via modulation of signalling proteins like Bcl-2.	Promotes parathyroid cell survival, leading to glandular enlargement and persistent hyperparathyroidism.

Individual variability in response to lithium therapy is influenced by genetic and epigenetic factors. Variations in HLA-DRB1 and DQB1, along with epigenetic modifications such as DNA methylation and histone changes, have been linked to lithium's therapeutic effects and inflammatory modulation [7].

Lithium may also induce the syndrome of irreversible lithium-effectuated neurotoxicity (SILENT), a condition characterized by persistent neurological deficits following acute lithium toxicity. SILENT occurs even within therapeutic lithium levels. At 1.5-2.5 mEq/L, patients exhibit altered mental states, and as levels increase to 2.5-3.5 mEq/L, symptoms such as confusion, disorientation, tachycardia, and hypotonia may develop. Levels exceeding 3.5 mEq/L can result in severe complications, including seizures and coma [8,9].

In this case, the patient exhibited features suggestive of nephrogenic diabetes insipidus (NDI), a condition commonly associated with chronic lithium use due to its downregulation of aquaporin-2 channels in the renal collecting ducts. However, persistent hypercalcemia can also impair renal concentrating ability and promote polyuria, likely through calcium-mediated tubular injury. Therefore, in patients like ours, who are exposed to both lithium and hypercalcemia, it is often not possible to definitively determine the primary etiology of NDI. It is likely that both mechanisms contributed synergistically, highlighting the importance of addressing both the lithium exposure and the underlying calcium disorder in management. NDI results from impaired aquaporin-2 activity due to lithium’s disruption of protein kinase A (PKA) signalling. While most patients maintain normal sodium levels due to intact thirst mechanisms, hypernatremia can occur in cases of inadequate fluid intake [10-12]. Bedford et al. highlight that chronic lithium exposure also predisposes patients to additional renal complications, including interstitial nephropathy, oxidative stress, mitochondrial dysfunction, and progressive renal failure [13]. Acute kidney injury and nephrotic syndrome, though rare, have also been reported in the literature [14,15].

Lithium’s activation of the Wnt signalling pathway promotes parathyroid cell proliferation, often resulting in hyperplasia rather than isolated adenomas. Approximately 40% of patients with lithium-induced hyperparathyroidism exhibit urinary calcium levels below 4 mmol/day, further complicating diagnosis. In two-thirds of cases, parathyroidectomy is not curative due to the underlying hyperplasia [16,17].

Calcimimetic agents can be useful for managing severe hypercalcemia when surgical intervention is not feasible. Haissaguerre and Vantyghem underline that hypercalcemia associated with lithium therapy becomes increasingly irreversible with prolonged exposure, limiting the likelihood of spontaneous recovery of parathyroid function after discontinuation [18]. However, in our clinical case, lithium discontinuation led to normalization of calcium levels, avoiding cincalcet.

Lithium’s impact on bone health remains controversial. Mechanistically, lithium has been associated with osteoblast activation through the Wnt/β-catenin/PI3K/Akt pathway and inhibition of osteoclast activity via RANK/RANKL/OPG signalling. However, in our case, lithium use was linked to reduced bone mineral density, highlighting a potential osteodamaging effect. This finding underscores the need for further investigation into the dual effects of lithium on bone metabolism, particularly in patients with concurrent osteoporosis or metabolic bone disorders [19,20]

## Conclusions

Addressing the limitations of our study, the challenge of deciding between surgical revision and conservative management for the detected adenoma remains uncertain and complex, particularly in psychiatric patients who may exhibit non-compliance and are prone to discontinuing follow-up care. This lack of adherence can hinder or underestimate the long-term outcomes of lithium use and the early detection of recurrences or complications. Furthermore, the generalizability of our findings is inherently limited, as this study focuses on a single case. This is particularly significant when considering the potential association of lithium with parathyroid carcinogenesis and osteoporosis, especially in the absence of calcimimetic therapy evaluation.
